# Efficacy and feasibility of Apatinib and S-1 as a novel oral induction therapy in locally advanced head and neck squamous cell carcinoma: an exploratory phase 2 open-label, single-arm trial

**DOI:** 10.3389/fonc.2023.1072538

**Published:** 2023-05-12

**Authors:** Wen Jiang, Rongrong Li, Lin Zhang, Shengjin Dou, Lulu Ye, Ziyang Shao, Sicheng Wu, Minjun Dong, Jiang Li, Guopei Zhu

**Affiliations:** ^1^ Department of Oral and Maxillofacial-Head and Neck Oncology, Shanghai Ninth People’s Hospital, Shanghai Jiao Tong University School of Medicine, College of Stomatology, Shanghai Jiao Tong University, National Center for Stomatology, National Clinical Research Center for Oral Diseases, Shanghai Key Laboratory of Stomatology, Shanghai Research Institute of Stomatology, Shanghai Center of Head and Neck Oncology Clinical and Translational Science, Shanghai, China; ^2^ Biostatistics Office of Clinical Research Center, Shanghai Ninth People’s Hospital, Shanghai Jiao Tong University School of Medicine, Shanghai, China; ^3^ Department of Radiology, Shanghai Ninth People’s Hospital, Shanghai Jiao Tong University School of Medicine, Shanghai, China; ^4^ Department of Oral Pathology, Shanghai Ninth People’s Hospital, College of Stomatology, Shanghai Jiao Tong University School of Medicine, Shanghai, China

**Keywords:** induction therapy, oral agents for cancer, locally advanced head and neck cancer, chemoradiotherapy, prospective and interventional study

## Abstract

**Objectives:**

The current standard nonsurgical treatment for locally advanced head and neck squamous cell cancer (LA-HNSCC) is concomitant chemoradiotherapy (CRT). Neoadjuvant chemotherapy combined with CRT has been explored in HNSCC patients and is an acceptable strategy. However, the occurrence of adverse events (AEs) restricts its application. We conducted a clinical study to explore the efficacy and feasibility of a novel induction therapy with orally administered apatinib and S-1 in LA-HNSCC.

**Materials and methods:**

This nonrandomized, single-arm, prospective clinical trial included patients with LA-HNSCCs. The eligibility criteria included histologically or cytologically confirmed HNSCC, with at least one radiographically measurable lesion detected by magnetic resonance imaging (MRI) or computerized tomography (CT) scan, age 18–75 years, and a diagnosis of stage III to IVb according to the 7^th^ edition of the American Joint Committee of Cancer (AJCC). Patients received induction therapy with apatinib and S-1 for three cycles (3 weeks/cycle). The primary endpoint of this study was the objective response rate (ORR) to induction therapy. The secondary endpoints included progression-free survival (PFS), overall survival (OS), and AEs during induction treatment.

**Results:**

From October 2017 to September 2020, 49 patients with LA-HNSCC were screened consecutively and 38 were enrolled. The median age of the patients was 60 years (range, 39-75). Thirty-three patients (86.8%) had stage IV disease according to the AJCC staging system. The ORR after induction therapy was 97.4% (95% confidence interval [CI]: 86.2%-99.9%). the 3-year OS rate was 64.2% (95% CI: 46.0%-78.2%) and 3-year PFS was 57.1% (95% CI: 40.8%-73.6%). The most common AEs during induction therapy were hypertension and hand-foot syndrome, which were manageable.

**Conclusion:**

Apatinib combined with S-1 as novel induction therapy for LA-HNSCC patients resulted in a higher-than-anticipated ORR and manageable adverse effects. With the associated safety profile and preferable oral administration route, apatinib combined with S-1 is an attractive exploratory induction regimen in outpatient settings. However, this regimen failed to show a survival benefit.

**Clinical trial registration:**

https://clinicaltrials.gov/show/NCT03267121, identifier NCT03267121.

## Introduction

Head and neck squamous cell carcinoma (HNSCC) is a group of tumors that originate in the oral cavity, oropharynx, pharynx and larynx. More than 500,000 new cases of HNSCC are diagnosed globally every year (1). Most HNSCC patients present with locally advanced disease at diagnosis. The current standard treatment for locally advanced HNSCC (LA-HNSCC) is concomitant chemoradiotherapy (CRT) or surgery followed by adjuvant (chemo)radiotherapy. An alternative approach involving induction chemotherapy (IC) remains controversial due to inconclusive evidence (2, 3). For instance, the PARADIGM trial did not observe significant superiority with IC compared to concurrent CRT in overall survival (OS) (4). Another phase III trial conducted in Spain did not observe a survival benefit of IC with CRT over CRT alone in patients with unresectable LA-HNSCC (5). However, the GORTEC 2007-02 phase III trial founded that for patients with very advanced HNSCC receiving three cycles of IC, there were no difference in progression-free survival (PFS) or OS but a significant difference in distant metastasis-free survival (6). Furthermore, the DECIDE trial showed that IC with CRT had a trend towards improved PFS compared to CRT alone in HNSCC patients with N2–N3 disease (7).

Based on results of TAX 323 and TAX 324 trials, TPF is the standard regimen for IC in clinical practice (2, 8, 9). Given the results of previous clinical trials investigating IC, treatment-related toxicities are the primary concern (10, 11). With this in mind, TPF regimen should be administered by experienced oncologists in hospitals to ensure patient safety and maximize adherence throughout treatment; however, this limits the application of IC (2). Moreover, severe AEs during IC could compromise the completion of subsequent chemoradiotherapy, leading to adverse impacts on survival. Therefore, a less toxic approach is worth developing.

Angiogenesis is a hallmark of tumor progression and is primarily mediated by the vascular endothelial growth factor (VEGF) pathway (12). Over 90% of patients with HNSCC express higher levels of VEGF and other angiogenic factors (13). Apatinib is a tyrosine kinase inhibitor (TKI) that selectively suppresses VEGFR-2, thereby inhibiting tumor angiogenesis (14). A preliminary investigation of apatinib has shown encouraging antitumor activity in several advanced solid tumors including HNSCC (15). Tegafur gimeracil oteracil (S-1) is an oral fluoropyrimidine anticancer agent comprising tegafur, 5-chloro-2, 4-dihydroxy pyridine, and potassium oxonate. A retrospective study demonstrated that S-1 can be administered in an outpatient setting to treat unresectable and distant metastatic HNSCC, with prolonged survival duration and acceptable toxicities (16). Antiangiogenic agents such as bevacizumab and sorafenib combined with chemotherapeutic agents have mostly been tested in recurrent or metastatic HNSCC (R/M HNSCC), resulting in mixed success (17–22). A large phase III trial demonstrated that bevacizumab combined with chemotherapy improved the response rate and PFS but did not improve OS in the first-line treatment setting of patients with R/M HNSCC (23). Given the current data on angiogenesis inhibitors and S-1 in HNSCC and that it is administered orally, which is preferable, we conducted a phase II trial to explore the efficacy and safety of induction treatment using apatinib with S-1 in LA-HNSCC patients.

## Patients and methods

### Study population and trial design

**Figure 1:**
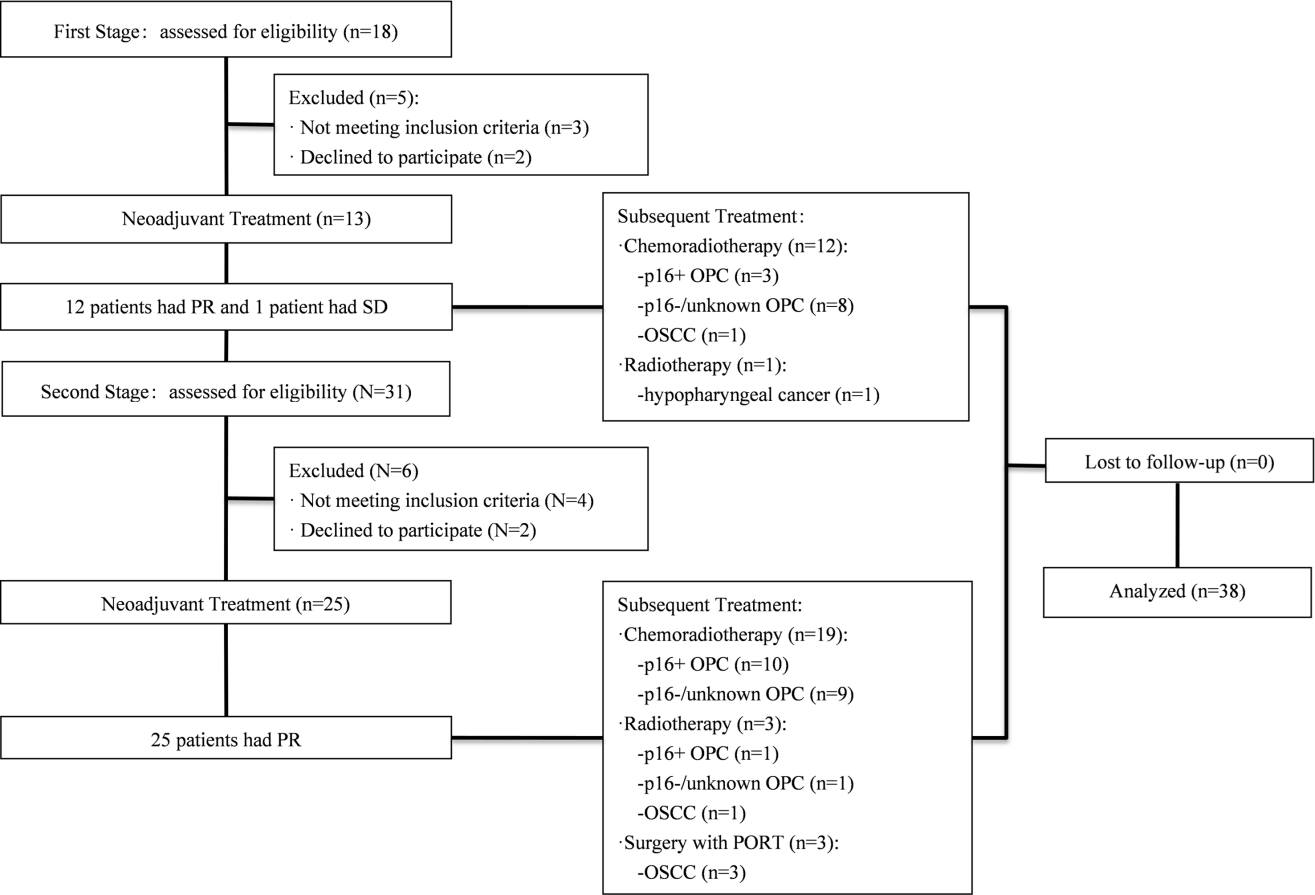
Flow diagram using Simon’s two-stage optimal design.

This was Simon’s two-stage, single-arm, prospective, phase II trial. The study protocol was approved by the Ethics Committee of Shanghai Ninth People’s Hospital and registered at Clinicaltrials.gov (ClinicalTrials. gov: NCT03267121). Patients with LA-HNSCC were recruited prospectively for this study (**Figure 1**). The eligibility criteria included histologically or cytologically confirmed HNSCC, with at least one radiographically measurable lesion detected by magnetic resonance imaging (MRI) or computerized tomography (CT) scan, age 18-75 years, a diagnosis of stage III to IVb according to the 7^th^ American Joint Committee of Cancer (AJCC), no prior chemotherapy or radiotherapy, Eastern Cooperative Oncology Group (ECOG) performance status score of 0-2, and normal organ and marrow function. All patients provided written informed consent before enrolling in the study.

### Induction therapy

Patients received induction therapy with apatinib and S-1 for three cycles (3 weeks/cycle). Apatinib was administered orally at a dose of 500 mg daily on days 1 to 21. S-1 was administered at 25mg/m^2^ twice daily for 14 consecutive days, followed by a 7-day break. Apatinib and S-1 were prescribed by each patient’s attending physician. Patients were required to record AEs in a dairy during treatment.

Treatment cycles were repeated if the absolute neutrophil count (ANC) was above 1.5x10^9^/L. Dose adjustments including interruptions and reductions were allowed for the management of treatment-related adverse events (TRAEs). There were two dose levels of apatinib (500 and 250 mg) and S-1 (25 and 20 mg/m^2^, twice daily). Generally, the doses of apatinib and S-1 were reduced in patients who developed grade 3 hematologic or grade 2 nonhematologic toxicities. Dose re-escalation in these patients was not permitted.

### Subsequent treatments

CRT was recommended within one week after induction therapy. Standard-fractionated radiotherapy was administered once daily for 6-7 weeks using intensity-modulated radiation therapy (IMRT) technique. Gross tumor volume (GTV) was determined based on radiographic findings before induction therapy. The total dose was 66-72 Gy in 2 Gy per fraction for gross disease. Clinical target volume (CTV) was defined as the GTV plus the areas considered at risk for containing microscopic disease delineated by the radiation oncologist. CTV_G represents GTV plus a margin of generally 5-10 mm. CTV_1 represented CTV_G plus the high-risk nodal regions and were treated to a dose of 60 Gy. CTV_2 represented low-risk nodal regions to receive elective irradiation with a dose of 50-54 Gy. A minimum margin of 3 mm around the CTV in all directions was required to define the planning target volume (PTV), except for situations in which the CTV was adjacent to the spinal cord or other critical normal tissues.

Triweekly cisplatin of 80mg/m^2^ was administered during the CRT. Nedaplatin was considered a suitable option at the discretion of the physicians. Salvage surgery was considered for residual disease after treatment. For patients with unresectable or borderline resectable oral cavity cancer (OCC), curative-intent surgery with postoperative radiotherapy is considered if the tumor was converted to a technically resectable disease.

### Assessments

The primary endpoint of this study was the objective response rate (ORR) to induction therapy. ORR was defined as the proportion of patients who had a partial response (PR) or complete response (CR) after induction therapy. Tumor response was evaluated within 7 days prior to the initiation of RT by an experienced radiologist according to the Response Evaluation Criteria in Solid Tumors (RECIST) guidelines, version 1.1 (24).

The secondary endpoints included PFS, OS, and AEs during induction treatment. PFS was calculated from the initiation of induction treatment to progression, second primary tumor, or death from any cause. For PFS, observations were censored if no event occurred. OS time was calculated from the initiation of induction treatment until death from any cause or censoring at the last contact. Tumor staging was performed according to the 7^th^ edition AJCC staging system for head and neck cancer.

AEs during the treatment were recorded and graded according to the National Cancer Institute Common Toxicity Criteria for Adverse Events version 5.0 (NCI-CTCAE v5.0). Oral mucositis during radiotherapy was assessed according to the Radiation Therapy Oncology Group (RTOG) criteria.

### Statistical analyses

Simon’s two-stage optimal design was used in this study (25). To detect an improvement ≥ 18% in the response rate compared to the TPF induction regimen with 80% power (one-sided p < 0.05), a sample size of 34 was required, which equates to an improvement in the ORR from 72% (observed in the TPF regimen) to 90% (2). Assuming a dropout rate of 10%, a total of 38 patients were required for this study. Thirteen patients were enrolled in the first stage. If there were at least 11 responses in these 13 patients, the study was stopped. Otherwise, 25 additional patients were recruited for a total of 38 patients.

All statistical analyses were performed using SPSS Statistics software (version 22.0; IBM SPSS Corp, Armonk, NY, USA). Descriptive statistics including mean, standard deviation, median, range, and percentage were used to describe the patient demographic, pathological, and clinical characteristics. Kaplan-Meier curves were used for the survival analysis. Reported p values were two-sided, and the significance level for all analyses was set at 0.05.

## Results

### Patient characteristics

Between October 2017 and September 2020, 49 patients with LA-HNSCCs were screened and 38 of them were enrolled (**Figure 1**). The baseline patient characteristics are summarized in **Table 1**. The median age of the patients was 60 years (range, 39-75). Thirty-three patients (86.8%) had stage IV disease. Twenty-seven patients (71.1%) had ten or more pack-years of tobacco history. The most common site of primary disease was the oropharynx (32 patients, 84.2%). Among the 32 patients with oropharyngeal cancers (OPCs), p16 immunohistochemical (IHC) testing was performed in thirty patients as a surrogate marker for HPV status. 16 patients were p16 negative and 14 patients were p16 positive (**Table 1**). Among the five patients with OCC, two were classified as having T4a disease and three were classified as having T4b disease, indicating that the tumors were borderline resectable (T4a) or unresectable (T4b) before induction therapy. The patient with hypopharyngeal cancer (HPC) presented with T4a disease. Among all patients, 21 patients (55.3%) presented with radiologic extranodal extension (ENE) on pretreatment as assessed by CT scan or MRI.

**Table 1:** Baseline characteristics.

Characteristic	All Patients (%) (n=38)	p16+ OPC Patients (%) (n=14)	Other Patients (%) (n=24)
Age, year			
Median (range)	60 (39–75)	55.5 (39-67)	60.5 (44-75)
Sex			
Male	31 (81.6)	11 (78.6)	20 (81.6)
Female	7 (18.4)	3 (21.4)	4 (81.6)
Primary Site			
Oropharynx	32 (84.2)	14 (100)	18 (75.0)
Oral cavity	5 (13.2)	–	5 (20.8)
Hypopharynx	1 (2.6)	–	1 (4.2)
T Stage*			
T2	6 (15.8)	4 (28.6)	2 (8.3)
T3	13 (34.2)	7 (50)	6 (25.0)
T4	19 (50.0)	3 (21.4)	16 (66.7)
N Stage*			
N0	1 (2.6)	0 (0)	1 (4.2)
N1	11(28.9)	4 (28.6)	7 (29.2)
N2a	8 (21.1)	3 (21.4)	5 (20.8)
N2b	10 (26.4)	4 (28.6)	6 (25.0)
N2c	7 (18.4)	2 (14.3)	5 (20.8)
N3	1 (2.6)	1 (7.1)	0 (0)
Total Stage*			
III	5 (13.2)	3 (21.4)	2 (8.3)
IVa-b	33 (86.8)	11 (78.6)	22 (91.7)
ECOG Score			
0	4 (10.5)	3 (21.4)	1 (4.2)
1	22 (57.9)	8 (57.1)	14 (58.3)
2	12 (31.6)	3 (21.4)	9 (37.5)
Smoking			
<10 pack-year	11 (28.9)	7 (50.0)	4 (16.7)
≥10 pack-year	27 (71.1)	7 (50.0)	20 (83.3)
P16 status of OPC (n=32)			
p16+	14 (43.8)	14 (100)	–
p16-	16 (50.0)	–	16 (88.9)
unknown	2 (6.2)	–	2 (11.1)
Subsite of OPC (n=32)			
Tonsillar	4 (12.5)	3 (21.4)	1 (5.6)
Base of tongue	13 (40.6)	6 (42.9)	7 (38.9)
Soft palate	10 (31.3)	2 (14.3)	8 (44.4)
Pharyngeal walls	5 (15.6)	3 (21.4)	2 (11.1)

OPC, oropharyngeal cancer; ECOG, Eastern Cooperative Oncology Group.

*Tumor staging was performed according to the 7^th^ edition AJCC staging system for head and neck cancer. "-" meaning N.A. (Not Applicable).

### Efficacy outcomes

**Figure 2:**
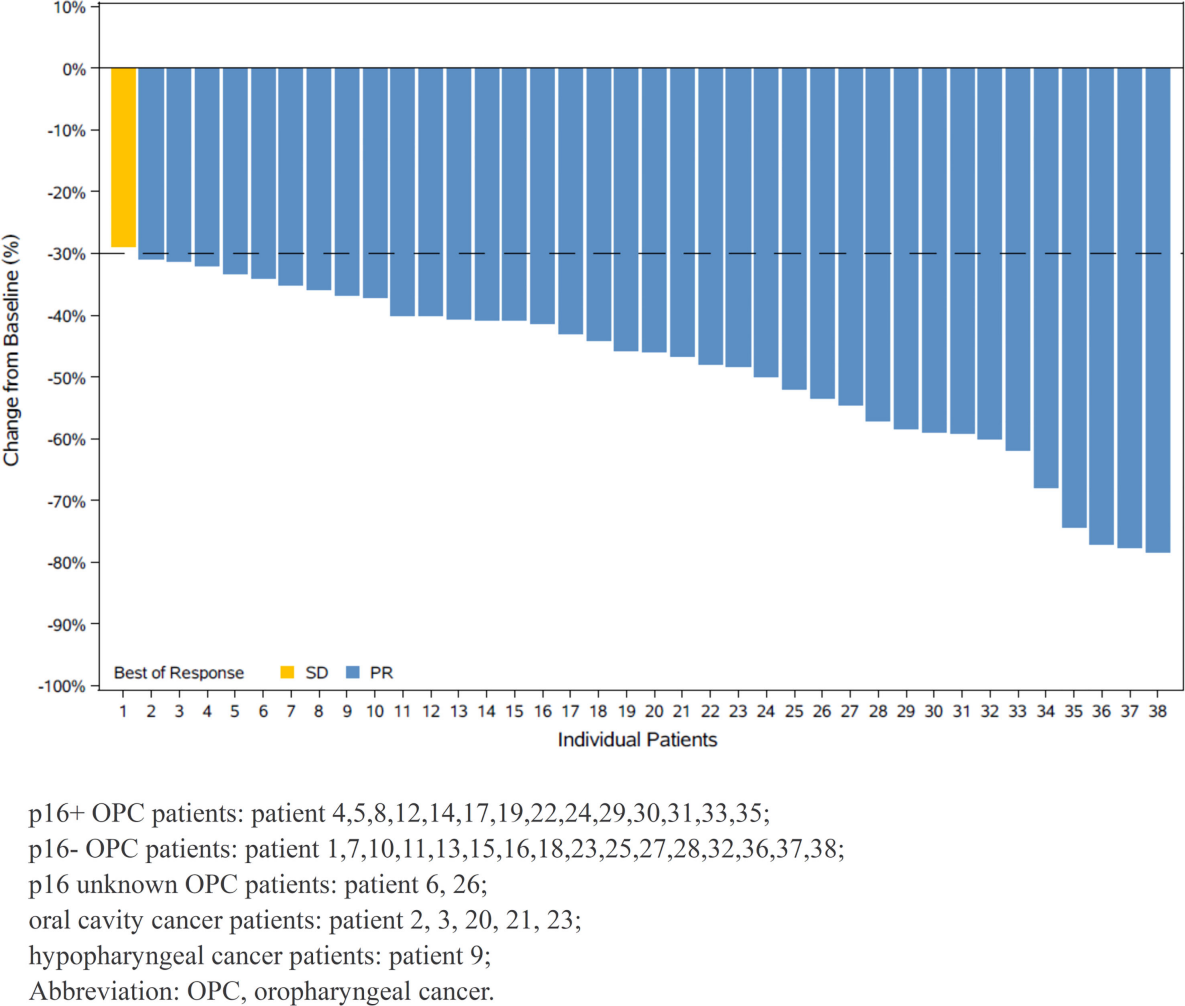
Waterfall plot of maximum percent change in tumor size from baseline as measured by Response Evaluatiuon Criteria in Solid Tumor (RECIST 1.1).

All 38 patients had measurable radiographic lesions before induction therapy. Thirty-seven patients (97.4%) were assessed as having PR, stable disease (SD) was noted in one patient (2.6%), and no progression disease (PD) was observed during neoadjuvant therapy. The ORR was 97.4% (95% confidence interval [CI]: 86.2-99.9%). All patients experienced tumor regression, and fourteen patients had a 50% or greater reduction in tumor size (**Figure 2**). The ORRs of p16+ OPC patients and other patients are listed in **Table 2**.

**Table 2:** Treatment responses and outcomes of patients.

	No. of All Patients (%)	No. of p16+ OPC Patients (%)	No. of Other Patients (%)
Response Rate after Induction Therapy (n=38)			
≥50%	>14 (36.8)	6 (42.9)	8 (33.3)
30%-50%	23 (60.5)	8 (57.1)	15 (62.5)
<30%	1 (2.6)	0 (0)	1 (4.2)
Subsequent Treatment after Induction Therapy (n=38)			
CRT	31 (81.6)	13 (92.9)	18 (75.0)
RT	4 (10.5)	1 (7.1)	3 (12.5)
Surgery with PORT	3 (7.9)	0 (0)	3 (12.5)
Outcomes after Induction Therapy and (Chemo)radiotherapy (n=35)			
CR	26 (74.3)	10 (83.3)	16 (69.6)
PR	9 (25.7)	2 (16.7)	7 (30.4)
Failure Patterns (n=15)			
LR	6 (37.5)	1 (33.3)	5 (38.5)
RR	5 (31.3)	1 (33.3)	4 (30.8)
LR and RR	2 (12.5)	0 (0)	2 (15.4)
DM	2 (12.5)	0 (0)	2 (15.4)
SPC	1 (6.3)	1 (33.3)	0 (0)

OPC, oropharyngeal cancer; CRT, chemoradiotherapy; RT, radiotherapy; PORT, postoperative radiotherapy; CR, complete response; PR, partial response; LR, local recurrence; RR, regional recurrence; DM, distant metastasis; SPC, second primary malignancy.

### Subsequent treatments after induction therapy

Among the 38 patients, 31 continued concurrent chemoradiotherapy after induction therapy. 4 patients received single-modality radiotherapy, and one patient with OPC was treated with radiotherapy because of the occurrence of grade 3 thrombocytopenia after induction therapy. The other three patients (two with OPC and one with HPC) refused concurrent chemotherapy due to relatively poor performance status and did not consider cetuximab because of the high associated costs. One patient underwent salvage neck dissection for a residual lymph node after radiotherapy, which resulted in a pathological complete response of the lymph node. Among the five patients with unresectable or borderline resectable OCC, three of them became resectable after induction therapy. Therefore, three patients with OCC underwent curative-intent surgery and postoperative radiotherapy (PORT).

Among the 31 patients who received definitive chemoradiotherapy, 29 completed planned cycles of concurrent chemotherapy. Two patients received only one cycle of chemotherapy because of the occurrence of grade 2 neutropenia and grade 3 oral mucositis (OM). All 38 patients (including three patients with PORT) were prescribed a dose of radiation without any unplanned breaks. Radiation therapy was delayed for 6 days in one patient due to grade 3 thrombocytopenia. The subsequent treatments for patients with/without p16+ OPCs are listed in **Table 2**.

Of 35 patients receiving (chemo)radiotherapy without surgery, 26 patients achieved a CR (74.3%), and 9 achieved a PR (25.7%) when evaluated one month after radiotherapy. Among the three OCC patients who underwent surgery, both radiological and pathological downstaging of the overall TNM stage was observed.

### Survival outcomes and failure patterns

**Figure 3:**
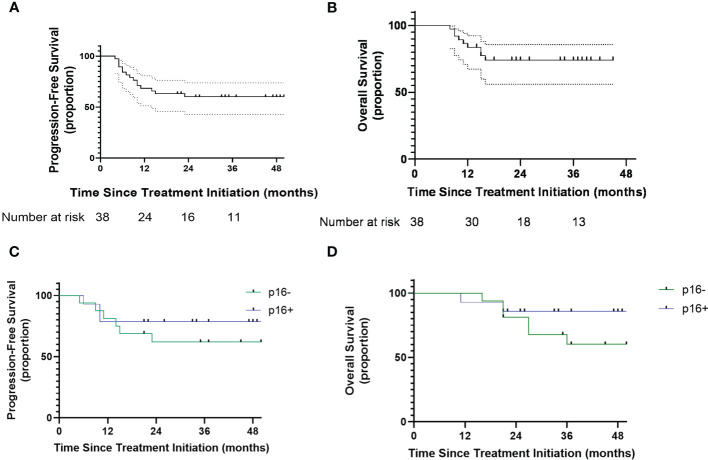
**(A)** Kaplan-Meier curves showing progression-free survival. **(B)** Kaplan Meier showing overall survival. **(C)** Kaplan Meier curves showing progression-free survival of oropharyngeal patients with different p16 status. **(D)** Kaplan-Meier curves showing overall survival of oropharyngeal patients with different p16 status. Number at risk = number at risk in 12-months increments.

All participants completed the follow-up. In the full intention-to-treat analysis set, the 3-year OS rate was 64.2% (95% CI: 46.0%-78.2%) and the 3-year PFS was 57.1% (95% CI: 40.8%-73.6%) (**Figures 3A, B**). With a median follow-up of 33 months (range, 8-56 months), median PFS and OS were not reached.

Thirteen patients had recurrences (six local, five regional, two local and regional), two patients experienced distant metastasis and one patient experienced the occurrence of a second primary cancer. Of the 13 patients with local-regional failure, 8 (34.7%) achieved 30%-50% tumor regression and 5 (35.7%) achieved ≥50% tumor regression. The failure patterns of p16+ OPC patients and other patients were listed in the **Table 2**. Six patients (46.2%) had local-regional recurrence within six months. Among the 6 patients that experienced rapid relapse, three received single-modality radiotherapy and the other three patients presented with OCC (two received definitive CRT). Seven patients with lymph node recurrence all presented with radiologic ENE before treatment.

Among the 32 patients with OPCs, the 3-year PFS rates of p16+ and p16- status were 78.6% and 61.8%, respectively (**Figure 3C**). The 3-year OS rates of p16+ and p16- status were 85.7% and 67.7%, respectively (**Figure 3D**).

### Safety and compliance

The median total treatment period of induction treatment was 55.5 days. Apatinib and S-1 were both reduced by one dose level in two patients because of grade 3 hand-foot syndrome (n=1) and stomach pain (n=1). Apatinib was reduced by one dose level but S-1 was continued in five patients with asymptomatic grade 3 hypertension (n=3), symptomatic grade 2 hypertension (n=1), or grade 2 tumor hemorrhage (n=1). Two patients discontinued induction therapy because of grade 3 thrombocytopenia (n=1) and symptomatic grade 3 hypertension (n=1).

The most common AE during induction treatment was hypertension (50%), followed by hand-foot syndrome (21.1%) and proteinuria (18.4%). The AEs during induction therapy and (chemo)radiotherapy are listed in **Table 3**.

**Table 3:** Hematologic and nonhematologic adverse events during induction therapy and (Chemo)radiotherapy.

Adverse Events	Patients(N=38)	
Adverse events during induction therapy		
Hematologic	Any grade, N (%)	Grade 3, N (%)
Neutropenia	0 (0)	0 (0)
Anemia	0 (0)	0 (0)
Thrombocytopenia	1 (2.6)	1 (2.6)
Elevated transaminase	5 (13.2)	0 (0)
Nonhematologic		
Hypertension	19 (50.0)	4 (10.5)
Hand-foot syndrome	8 (21.1)	1 (2.6)
Proteinuria	7 (18.4)	0 (0)
Pharyngolaryngeal pain	5 (13.2)	0 (0)
Stomach pain	3 (7.9)	1 (2.6)
Fatigue	4 (10.5)	0 (0)
Hemorrhage	3 (7.9)	0 (0)
Oral pain	2 (5.3)	0 (0)
Oral mucositis	2 (5.3)	0 (0)
Adverse events during (chemo)radiotherapy		
Hematologic	Any grade, N (%)	Grade 3, N (%)
Neutropenia	12 (31.6)	0 (0)
Anemia	25 (65.8)	0 (0)
Thrombocytopenia	1 (2.6)	0 (0)
Elevated transaminase	3 (7.8)	0 (0)
Nonhematologic		
Anorexia	16 (42.1)	3 (7.8)
Dysphagia	33 (86.8)	11 (28.9)
Oral mucositis	38 (100)	8 (21.1)
Fatigue	26 (68.4)	4 (10.5)
Nausea	26 (68.4)	2 (5.3)
Vomiting	23 (60.5)	1 (2.6)
Weight loss	34 (89.5)	3 (7.9)

The most common type of nonhematologic toxicity during definitive (chemo)radiotherapy or postoperative radiotherapy was oral mucositis. Eight patients (21.1%) had grade 3 oral mucositis. None of the patients experienced newly appearing hematologic AEs that exceeded grade 3. All patients received the prescribed RT dose without unplanned RT breaks. No treatment-related deaths occurred during this period.

## Discussion

To our knowledge, this is the first prospective trial to explore the efficacy and safety of apatinib plus S-1 as induction therapy for LA-HNSCC patients. Our study showed a favorable ORR of 97.4% (95% CI: 86.2%-99.9%) with this regimen. Toxic effects were manageable and no treatment-related deaths were observed.

Induction therapy followed by CRT was shown to be noninferior and could decrease distant metastatic progression compared to CRT alone, especially in high-risk groups (2, 3, 26). IC is observed to shrink tumors, eradicate micrometastasis and preserve organs in clinical practices. In previous clinical trials, the ORRs of IC with the TPF regimen ranged between 54% and 76% (4, 7, 8, 26). In this study, the radiologic response rate of 97.4% was comparatively higher. Furthermore, at least a 50% decrease in tumor size was found in 14 patients (36.8%) in the current study. Therefore, this regimen is advantageous for quickly shrinking tumors. In addition to other medical reasons, IC is practical for controlling tumor growth in patients receiving care at high-volume institutions and when prompt surgery cannot be arranged in a timely manner or during the design of a radiotherapy plan (27, 28). This is especially true for OCCs that grow rapidly. Our study included five OCC patients with T4 tumors, and our data support the possibility of using induction therapy to rapidly shrink tumors. For three OCC patients who received curative-intended surgery after induction therapy, the pathological downstaging rate was 100% and two patients were alive without tumor progression after two years. A retrospective study also suggested that cisplatin-based IC played a role in converting borderline resectable disease or definitively unresectable disease to a technically resectable disease in OCCs, which may enable resection and subsequently improved outcomes (29). In contrast, Gangopadhyay et al. demonstrated that surgery after downstaging with IC had a similar survival as compared to upfront surgery for OCC patients (30). Using IC to enable resection remains controversial due to a lack of sufficient evidence supporting this approach and more randomized studies are required before induction therapy is incorporated into the treatment algorithm of OCCs. The induction therapy regimen used in the current study might be promising for patients with advanced disease. Moreover, the oral route allows this approach to be easily administered in outpatient settings,

Despite a relatively high ORR, this study failed to show a favorable survival rate in contrast to previous studies. In the TAX 324 study, IC with three cycles of TPF followed by CRT resulted in a 3-year OS of 62% (8). The majority of patients in this study had OPCs, and 16 patients were p16 negative. HPV-associated OPC appears to respond better to treatment and has a better prognosis than HPV-negative OPC (31). However, the survival rate of our study was not comparable to that reported in other clinical trials with a high proportion of OPC. This may be due to the relatively high proportion (46.9%) of nontonsillar oropharyngeal subsites that were enrolled in this study. One study demonstrated that, even in cases of HPV-positive disease, patients with nontonsillar oropharyngeal subsites had an approximately 3-year cause-specific survival of 60%, which is worse than other subsites (32). Marklund et al. suggested that p16 status should only be evaluated in cases of tonsillar and base of tongue squamous cell carcinomas (33). The 3-year OS was approximately 60% in p16-positive OPC patients of the soft palate and pharyngeal walls (33). It is important to note that most patients in this trial showed lymph node metastasis with ENE. A retrospective study showed that HPV-associated OPCs with radiologic ENE have a higher risk of distant metastasis and reduced survival (34). Another study showed that the addition of cisplatin-based systemic agents did not negate the distant metastasis rate or improve loco-regional control in radiologic ENE oropharyngeal cancer (35). In our study, 16 of 32 OPC patients were diagnosed with radiologic ENE and only one patient developed distant metastasis. Also, six patients experienced local-regional failure within six months. Among these six patients, three received radiotherapy instead of standard CRT, which would have compromised the survival outcomes. The other three patients with rapid relapses had T4 oral cavity diseases, and two were treated with CRT. One OCC patient with borderline resectable disease underwent surgery and PORT after induction therapy. The rapid relapse of those patients might be associated with the unsatisfactory oncologic outcomes observed in this study. These results indicate that standard chemoradiotherapy helped OPC patients achieve satisfactory survival outcomes.

Treatment-associated toxicities of intravenous chemotherapy remain a major concern. Toxic death rates ranged from 2% to 7% in various trials using the TPF regimen (10). The CONDOR study was prematurely terminated because only 32% of patients could receive the planned dose of cisplatin during CRT due to toxicities associated with induction TPF (11). The toxic effects of IC can interfere with the completion of subsequent chemoradiotherapy, which has deleterious effects on local control and survival (36). In this study, fewer toxic effects were observed during induction treatment with apatinib and S-1 than cisplatin-based IC, and no severe adverse events were reported. A major issue regarding the use of antiangiogenic therapy in HNSCC is bleeding events (21, 37). In our study, only three patients experienced mild and controllable bleeding, indicating that anti-VEGF therapy can be used safely in treatment-naive HNSCC patients. A pilot phase I trial also demonstrated that neoadjuvant treatment using apatinib and anti-programmed cell death-1 (PD-1) monotherapy was well tolerated for OCC patients (38). S-1 has shown activities in advanced and recurrent/metastatic HNSCC with relatively mild toxic effects (39). A case report showed that the combined administration of apatinib and S-1 resulted in partial responses in advanced HNSCC patients, while only mild toxicities were observed (18). A retrospective study also demonstrated that apatinib combined with S-1 had mild and tolerable toxicities for metastatic nasopharyngeal cancer patients (40). Safety profiles reported in those studies were in consistent with our results. Furthermore, since cisplatin was not used in this regimen, there were fewer overlapping toxicities with subsequent cisplatin-based chemoradiotherapy, enabling it to be safely incorporated with existing treatment modalities for patients with HNSCCs.

This study has several limitations. First, this was a single-arm trial without controls, and selection bias could not be ruled out. Another limitation is that this study recruited a large proportion of OPCs. In recent years, the importance of HPV as a prognostic marker for head and neck cancer has been recognized. However, p16/HPV testing was not a routine practice in our hospital until early 2018 for practical reasons. Therefore, p16 IHC testing was performed in 30 of 32 OPC patients as a supplementary test, and tissue specimens were not available for two patients. Last, the sample size was relatively small.

In conclusion, apatinib with S-1 as induction therapy exhibited a high ORR with relatively minor toxicities in patients with LA-HNSCC. With the associated safety profile and preferable oral administration route, this combination is an attractive exploratory induction regimen for outpatient settings. However, this regimen failed to show a survival benefit.

## Data availability statement

The raw data supporting the conclusions of this article will be made available by the authors, without undue reservation.

## Ethics statement

The studies involving human participants were reviewed and approved by Ethics Committee of Shanghai Ninth People’s Hospital. The patients/participants provided their written informed consent to participate in this study.

## Author contributions

GZ designed the study. WJ was the main author of the manuscript and was involved in data collection and analysis. JL confirmed the pathological diagnosis of each patient. LZ and ZS were involved in patients’ anti-cancer treatments. SD and LY helped collect data. SW conducted the statistical analysis and interpretation of the results. GZ and RL supervised the project and revised the manuscript. All authors contributed to the article and approved the submitted version.
